# Mortality in the JACC Study till 1999

**DOI:** 10.2188/jea.15.S74

**Published:** 2005-05-16

**Authors:** Yoshiyuki Watanabe, Kotaro Ozasa, Junko Nagura, Kyohei Hayashi, Takesumi Yoshimura, Akiko Tamakoshi

**Affiliations:** 1 Department of Epidemiology for Community Health and Medicine, Kyoto Prefectural University of Medicine Graduate School of Medical Science.; 2 Institute of Industrial Ecological Sciences, University of Occupational and Environmental Health.; 3 Department of Preventive Medicine/Biostatistics and Medical Decision Making, Nagoya University Graduate School of Medicine.; 4 See acknowledgement for the present investigator involved in the JACC Study.

**Keywords:** Japan Collaborative Cohort Study (JACC Study), Cancer, Mortality

## Abstract

BACKGROUND: We have been conducting a cohort study named “the Japan Collaborative Cohor Study (JACC Study) for Evaluation of Cancer Risk sponsored by the Ministry of Education, Science, Sports and Culture of Japan (Monbusho)” since 1988. The aim of this paper is to describe the mortality of our JACC cohort in the follow-up period from 1988 through 1999, to compare it with the mortality, especially cancer deaths, of the Japanese population in the same period and to compare the causes of mortality by district among the cohort.

METHODS: We conducted a follow-up study of 110,792 Japanese inhabitants aged 40-79 years in 1988-1990 for about 10 years to the end of 1999.

RESULTS: Of 46,465 males, 37,750 (81.2%) were alive, 7,238 (15.6%) were dead and 1,477 (3.2%) had moved out of the study areas. The figures were 57,016 (88.6%), 4,940 (7.7%) and 2,371 (3.7%) among 64,327 females, respectively. The mean follow-up period was 9.9 years. The proportion of cancer deaths by site in our cohort members was almost same as the Japanese population aged 40-79 years old in 1995. Sex-specific standardized mortality ratios of total deaths, all cancer deaths, and most cancers in our cohort were less than 100 in both males and females for total cohort and the cohort by district.

CONCLUSION: Our cohort members appeared to be almost the same or slightly healthier and less likely to die from total causes and cancers than the general population.

A large-scale population based cohort study named “the Japan Collaborative Cohort Study (JACC Study) for Evaluation of Cancer Risk sponsored by the Ministry of Education, Science, Sports and Culture of Japan (Monbusho)”^1^ was initiated in 1988. The purpose of this study is to address the relationship between recent Japanese lifestyles and cancer. There were no other cohort studies in Japan at that time since Hirayama’s large-scale cohort study^2^ on cancer which was initiated in 1965 and terminated in 1982, even though the Japanese lifestyle, especially dietary habits, have dramatically changed since the end of the Second World War in 1945.^3^ Epidemiologic studies using questionnaires on smoking, drinking and diet are important. However, those using biological markers can provide much more informative evidence for cancer pathogenesis. Therefore, another purpose of the JACC Study is to investigate the relationship between biological markers and cancer risk.

This paper will aim to describe the mortality of our JACC cohort in the follow-up period from 1988 through 1999, to compare it with the mortality, especially cancer deaths, of the Japanese population in the same period and to compare the causes of mortality by district among the cohort.

## METHODS

The JACC study was conducted in 45 areas from 19 prefectures throughout Japan; 3 towns in Hokkaido district, 5 towns in Tohoku district, 5 towns in Kanto district, one city, 3 towns and 2 villages in Chubu district, 8 towns and 2 villages Kinki district, one city and one town in Chugoku district, and 4 cities, 9 towns and one village in Kyushu district. A basic cohort population of 127,477 healthy inhabitants (54,032 males and 73,445 females) in the above areas responded to the baseline questionnaire in 1988-1990. Basic cohort members, including 46,465 men and 64,327 women (110,792 in total) aged 40-79 years at entry, were followed up for about 10 years to the end of 1999.

The follow-up survey was conducted using population registries in local municipalities to determine the vital and residential status of the cohort in each area. All the cases that moved out of the study areas were treated as censored cases. Five subjects who were expunged from their residence record by authorities were also treated as censored cases and included in cases that moved out for computing the cohort numbers by follow up status as of end of 1999. All deaths that occurred in the cohort were ascertained by death certificates from local public health centers in the study areas under the authorities’ permission from the Director-General of the Prime Minister’s Office (Ministry of Public Management, Home Affairs, Post, and Telecommunications). The underlying causes of death were coded according to the International Classification of Diseases and Injuries (ICD) 10th version by verifying computer-stored data in the Ministry of Health, Labour and Welfare with the permission. Those already coded according to the ICD 9th version (from the time of the baseline survey through 1994) and stored in the computer data-base in the Ministry of Health and Welfare were re-coded in 1999 according to the ICD 10th version (after 1995), using a specifically developed computer program^4^ for converting the ICD 9th code to the 10th.

Follow-up condition (alive, dead, or moved) by sex and age group at entry as of the end of 1999 was computed. For those dead, causes of death, especially of cancer deaths, by sex and age group at entry as of the end of 1999 were also computed. Sex-specific standardized mortality ratios (SMRs) were calculated using sex- and age-specific person-years of following-up and sex- and age-specific mortality rates for all Japan in 1988-1999.^5^ From a practical reason, the mortality rates in 1989 were used for the follow-up data in 1988-1990, and by the same manner, the rates in 1992, 1995, and 1998 were used for each three years of follow-up. Confidence intervals of SMR were calculated according to chi-square distribution when the observation number was 10 or larger,^6^ and according to Poisson distribution when less than 10.^7^

Our entire study design, which comprised singular and collective use of epidemiologic data and biological materials (serum only), was approved in 2000 by the Ethical Board at Nagoya University School of Medicine, where the central secretariat of the JACC study is located.

## RESULTS

Table 1 shows the age distribution of the cohort members aged 40-79 years at the time of their enrolment in the study and their follow-up condition as of the end of 1999. Of 46,465 males, 37,750 (81.2%) were alive, 7,238 (15.6%) were dead, and 1,477 (3.2%) had moved out of the study areas. The figures were 57,016 (88.6%), 4,940 (7.7%), and 2,371 (3.7%) among 64,327 females, respectively. The mean follow-up period was about 10 years.

**Table 1.** Age distribution of cohort members at entry and deaths/move-outs as of the end of 1999.

	Age at entry (year)								

	40-44	45-49	50-54	55-59	60-64	65-69	70-74	75-79	Total
Male									
Total	6,002	5,806	6,322	7,695	8,429	5,518	4,024	2,669	46,465
Alive	5,518	5,331	5,689	6,725	6,917	4,079	2,334	1,157	37,750
(%)	91.9	91.8	90.0	87.4	82.1	73.9	58.0	43.4	81.2
Dead	147	219	441	785	1337	1306	1572	1431	7238
(%)	2.5	3.8	7.0	10.2	15.9	23.7	39.1	53.6	15.6
Moved	337	256	192	185	175	133	118	81	1477
(%)	5.6	4.4	3.0	2.4	2.1	2.4	2.9	3.0	3.2

Female									
Total	7,557	7,926	9,108	10,816	11,114	8,602	5,557	3,647	64,327
Alive	7,074	7,485	8,565	10,094	10,092	7,364	4,189	2,153	57,016
(%)	93.6	94.4	94.0	93.3	90.8	85.6	75.4	59.0	88.6
Dead	83	136	236	425	713	944	1099	1304	4940
(%)	1.1	1.7	2.6	3.9	6.4	11.0	19.8	35.8	7.7
Moved	400	305	307	297	309	294	269	190	2371
(%)	5.3	3.9	3.4	2.8	2.8	3.4	4.8	5.2	3.7

Total cancer deaths accounted for 38.7% (2,798) and 35.0% (1,730) in male and female total deaths (7,238 and 4,940) as shown in Table 2. Among male cancer deaths, cancers of the lung (code of ICD 10th: C33, C34), stomach (C16), and liver (C22) were the three commonest sites, accounting for 23.0%, 20.8%, and 13.1%, respectively. In women, the three commonest sites were cancers of the stomach, lung, and liver, accounting for 16.6%, 11.8%, and 9.3%, respectively. The commonest were the stomach, large intestine (12.3%), and lung when the large intestine was defined by the cancer of colon and rectum (C18-20).

**Table 2.** Age distribution of total deaths, all cancer deaths, and site-specific cancer deaths as of the end of 1999.

	Age at entry (year)								

	40-44	45-49	50-54	55-59	60-64	65-69	70-74	75-79	Total
Total deaths	230	355	677	1,210	2,050	2,250	2,671	2,735	12,178
All cancer deaths	95	154	323	578	1,008	850	845	675	4,528
%	41.3	43.4	47.7	47.8	49.2	37.8	31.6	24.7	37.2

Male									

Total deaths	147	219	441	785	1,337	1,306	1,572	1,431	7,238
Cancer deaths	50	88	194	365	677	515	509	400	2,798
%	34.0	40.2	44.0	46.5	50.6	39.4	32.4	28.0	38.7

Lung(C33,C34)	11	11	37	62	174	136	120	93	644
Stomach(C16)	13	19	36	84	130	109	109	82	582
Liver(C22)	5	12	42	62	104	52	56	33	366
Pancreas(C25)	3	5	9	17	35	30	32	30	161
Colon(C18)	4	5	15	14	32	23	27	24	144
Gall bladder/bile duct(C23,C24)	1	4	5	23	32	34	21	20	140
Rectum(C19,C20)	3	8	9	27	18	19	20	19	123
Esophagus(C15)	4	5	12	19	29	18	14	7	108
Prostate(C61)	0	0	1	4	13	20	33	32	103
Malignant lymphoma(C81-C85)	0	3	6	9	18	13	6	8	63
Mouth(C00-C14)	1	1	0	9	17	8	4	9	49
Bladder(C67)	0	1	0	4	13	5	10	6	39
Lalynx(C32)	0	0	1	1	2	2	3	1	10
Skin(C43,C44)	0	0	0	2	1	0	0	2	5
Others	5	14	21	28	59	46	54	34	261

Female									

Total deaths	83	136	236	425	713	944	1,099	1,304	4,940
Cancer deaths	45	66	129	213	331	335	336	275	1,730
%	54.2	48.5	54.7	50.1	46.4	35.5	30.6	21.1	35.0

Stomach(C16)	7	10	14	43	51	58	56	48	287
Lung(C33,C34)	6	3	15	24	44	40	43	30	205
Liver(C22)	1	4	8	22	33	49	21	23	161
Pancreas(C25)	1	5	10	17	35	29	34	25	156
Colon(C18)	0	5	12	17	23	34	26	34	151
Gall bladder/bile duct(C23,C24)	3	9	9	15	20	27	38	27	148
Breast(C50)	9	13	14	16	17	11	10	4	94
Uterus(C53-C55)	6	2	12	9	12	11	13	14	79
Rectum(C19,C20)	2	4	5	4	19	8	13	7	62
Malignant lymphoma(C81-C85)	1	1	4	10	10	7	10	4	47
Ovary(C56)	4	4	9	6	17	5	6	5	56
Esophagus(C15)	0	1	2	1	2	6	5	6	23
Bladder(C67)	0	0	0	1	4	3	7	6	21
Mouth(C00-C14)	0	0	2	3	1	2	6	5	19
Skin(C43,C44)	0	0	1	2	1	0	1	3	8
Lalynx(C32)	0	0	0	0	1	1	0	0	2
Others	5	5	12	23	41	44	47	34	211

in the parentheses: code of ICD 10th

Sex-specific SMRs of total deaths, all cancer deaths, and site-specific cancer deaths were shown in Table 3. Site-specific SMRs for a total cohort were less than 100 except for cancer of the gall bladder/bile duct (C23, C24) in males and pancreas (C25) and skin (C43, C44) in females. SMRs of total deaths and all cancer deaths by district were less than 100 in both males and females. Most of the SMRs by cancer site and district were less than 100 though some exceeded this.

**Table 3.** Sex-specific satandardized mortality raios (SMRs) and 95% confidence intervals (CI) of total deaths, all cancer deaths, and site-specific cancers by district.

	Hokkaido	Tohoku	Kanto	Chubu	Kinki	Chugoku	Kyushu	Total
	SMR95%CI	SMR95%CI	SMR95%CI	SMR95%CI	SMR95%CI	SMR95%CI	SMR95%CI	SMR95%CI
Male								
Total dealth	61 ( 52, 72)	84 ( 79, 89)	77 ( 71, 83)	75 ( 72, 78)	80 ( 75, 84)	79 ( 75, 84)	84 ( 79, 90 )	78 ( 76, 80 )
All Cancer death	72 ( 57, 90)	87 ( 78, 96)	76 ( 66, 87)	73 ( 68, 78)	82 ( 75, 90)	83 ( 75, 92)	97 ( 87, 106 )	81 ( 78, 84 )
Cancer site:								
Lung (C33,C34)*	95 ( 59, 145)	102 ( 83, 124)	83 ( 62, 109)	73 ( 63, 85)	115 ( 97, 136)	78 ( 62, 97)	84 ( 66, 105 )	87 ( 81, 94 )
Stomach (C16)	62 ( 33, 106)	82 ( 65, 103)	100 ( 76, 130)	86 ( 75, 89)	80 ( 64, 98)	64 ( 48, 82)	96 ( 77, 119 )	83 ( 77, 90 )
Liver (C22)	34 ( 11, 79)	61 ( 43, 83)	64 ( 41, 94)	56 ( 45, 69)	58 ( 43, 78)	129 ( 101, 161)	150 ( 121, 185 )	78 ( 70, 87 )
Pancreas (C25)	120 ( 48, 247)	115 ( 77, 165)	82 ( 44, 141)	63 ( 45, 86)	97 ( 66, 138)	86 ( 54, 131)	78 ( 47, 122 )	84 ( 71, 98 )
Colon (Cl8)	78 ( 25, 182)	57 ( 33, 93)	28 ( 9, 65)	75 ( 56, 98)	45 ( 26, 73)	83 ( 53, 124)	94 ( 61, 139 )	67 ( 57, 79 )
Gall bladder/bile duct (C23,24)	125 ( 41, 291)	129 ( 82, 194)	145 ( 82, 234)	104 ( 76, 138)	71 ( 41, 115)	110 ( 68, 168)	71 ( 37, 124 )	102 ( 86, 121 )
Rectum (C19,C20)	23 ( 1, 128)	97 ( 57, 153)	59 ( 24, 122)	92 ( 66, 123)	81 ( 48, 126)	71 ( 38, 122)	122 ( 77, 185 )	87 ( 72, 104 )
Esophagus (Cl5)	101 ( 33, 235)	118 ( 77, 175)	30 ( 8, 77)	59 ( 40, 83)	41 ( 21, 74)	69 ( 38, 116)	88 ( 52, 139 )	68 ( 56, 82 )
Prostate (C61)	95 ( 20, 277)	103 ( 58, 169)	65 ( 24, 142)	97 ( 68, 134)	58 ( 29, 105)	109 ( 65, 170)	89 ( 46, 154 )	90 ( 73, 109 )
Malignant lymphoma (C81-C85)	130 ( 27, 380)	79 ( 34, 156)	94 ( 34, 205)	71 ( 42, 112)	70 ( 32, 133)	97 ( 47, 179)	93 ( 43, 177 )	82 ( 63, 105 )
Mouth (C00-C14)	57 ( 1, 317)	147 ( 73, 262)	63 ( 13, 184)	48 ( 22, 91)	64 ( 23, 140)	170 ( 87, 295)	99 ( 40, 204 )	87 ( 65, 115 )
Bladder (C67)	65 ( 2, 362)	57 ( 16, 146)	114 ( 37, 266)	44 ( 19, 87)	89 ( 38, 175)	63 ( 20, 147)	120 ( 52, 236 )	71 ( 51, 97 )
Lalynx (C32)	0 —	0 —	0 —	77 ( 25, 179)	62 ( 8, 224)	37 ( 1, 206)	81 ( 10, 292 )	51 ( 24, 94 )
Skin (C43, C44)	0 —	196 ( 24, 708)	0 —	38 ( 1, 212)	77 ( 2, 429)	0 —	100 ( 3, 557 )	63 ( 20, 147 )

Female								
Total dealth	66 ( 55, 79)	72 ( 67, 78)	71 ( 64, 79)	69 ( 65, 72)	69 ( 64, 74)	61 ( 57, 65)	74 ( 68, 79 )	69 ( 67, 70 )
All Cancer death	75 ( 55, 99)	79 ( 69, 89)	75 ( 63, 89)	80 ( 74, 87)	63 ( 55, 72)	72 ( 64, 80)	83 ( 74, 93 )	76 ( 72, 79 )
Cancer site:								
Stomach (C16)	28 ( 6, 82)	80 ( 57, 110)	91 ( 59, 133)	79 ( 63, 97)	73 ( 52, 99)	58 ( 41, 79)	93 ( 70, 122 )	76 ( 67, 85 )
Lung (C33,C34)	52 ( 14, 133)	63 ( 40, 96)	99 ( 61, 151)	65 ( 49, 85)	60 ( 39, 89)	87 ( 64, 117)	89 ( 62, 124 )	74 ( 64, 85 )
Liver (C22)	82 ( 27, 191)	86 ( 54, 128)	50 ( 22, 99)	70 ( 51, 95)	35 ( 18, 63)	86 ( 59, 120)	133 ( 95, 183 )	78 ( 66, 91 )
Pancreas (C25)	134 ( 49, 292)	89 ( 53, 140)	106 ( 56, 181)	135 ( 104, 172)	75 ( 44, 118)	67 ( 41, 103)	74 ( 43, 119 )	97 ( 82, 113 )
Colon (Cl8)	137 ( 59, 270)	111 ( 74, 160)	75 ( 39, 132)	75 ( 55, 100)	55 ( 32, 88)	61 ( 39, 91)	54 ( 31, 87 )	73 ( 62, 85 )
Gall bladder/bile duct (C23,24)	131 ( 48, 286)	80 ( 46, 128)	124 ( 71, 201)	90 ( 66, 120)	82 ( 51, 125)	67 ( 42, 102)	83 ( 50, 128 )	86 ( 73, 101 )
Breast (C50)	79 ( 21, 202)	51 ( 24, 93)	50 ( 18, 109)	64 ( 42, 93)	45 ( 21, 82)	98 ( 61, 150)	68 ( 39, 110 )	64 ( 52, 79 )
Uterus (C53-C55)	0 —	72 ( 33, 137)	80 ( 29, 174)	71 ( 43, 110)	104 ( 58, 171)	98 ( 56, 159)	89 ( 47, 151 )	82 ( 65, 102 )
Rectum (C19,C20)	75 ( 9, 271)	94 ( 47, 170)	57 ( 16, 146)	56 ( 31, 93)	29 ( 8, 74)	81 ( 43, 139)	96 ( 51, 165 )	68 ( 52, 87 )
Ovary (C56)	37 ( 1, 206)	27 ( 6, 79)	61 ( 17, 156)	113 ( 75, 165)	32 ( 9, 82)	84 ( 42, 150)	48 ( 18, 105 )	68 ( 52, 89 )
Malignant lymphoma (C81-C85)	197 ( 41, 575)	58 ( 16, 148)	24 ( 1, 134)	69 ( 34, 123)	123 ( 58, 224)	109 ( 54, 195)	90 ( 36, 185 )	86 ( 63, 114 )
Esophagus (Cl5)	0 —	80 ( 16, 234)	88 ( 11, 318)	78 ( 31, 161)	156 ( 63, 321)	54 ( 11, 158)	23 ( 1, 128 )	76 ( 48, 115 )
Bladder (C67)	0 —	73 ( 9, 264)	117 ( 14, 422)	128 ( 59, 243)	29 ( 1, 162)	65 ( 13, 190)	127 ( 35, 325 )	90 ( 56, 138 )
Mouth (C00-C14)	0 —	211 ( 77, 460)	0 —	107 ( 43, 220)	89 ( 18, 260)	50 ( 6, 181)	31 ( 1, 173 )	85 ( 51, 133 )
Skin (C43, C44)	0 —	0 0 0	0 —	288 ( 106, 628)	190 ( 23, 686)	0 —	0 —	113 ( 49, 223 )
Lalynx (C32)	0 —	404 ( 10,2250)	0 —	0 —	335 ( 8, 1866)	0 —	0 —	99 ( 12, 357 )

* Code in parenthesis is ICD-10.

## DISUCUSSION

The follow-up condition of cancer deaths by site as of the end of 1999 was almost same as of the end of 1997.^1^ The three commonest sites, cancers of the lung, stomach, and liver in males were of the same order and almost the same proportion among all cancer deaths as of the end of 1997.^1^ Among females, the three commonest sites, cancers of the stomach, large intestine, and lung were same in each site as of the end of 1997,^1^ but the proportion of cancer of the large intestine (12.3%) exceeded that of the lung (11.8%) if the cancer of the colon and rectum were combined.

SMRs of total deaths, all cancer deaths, and most site-specific cancers were less than 100 in both males and females. This means that our cohort members appeared to be less likely to die from total causes and cancers in comparison with the Japanese population as observed other Japanese cohort.^8^ Because some of the cohort members were selected from participants in health check-ups or other kinds of screening, they might have had slightly healthier lifestyles that prevented them from dying with lifestyle related diseases such as cancers and cerebrovascular diseases. Cohort members of each district also appeared to be slightly healthier than the general population, as most SMRs of total deaths and site-specific cancer deaths by district were less than 100. It might be due to the small cohort size that some SMRs of site-specific cancer deaths by district were more than 100. Even though our cohort members were slightly healthier than the general Japanese population in the study period, internal comparisons between an exposed group and a group of unexposed to any factors within the cohort can also be justified as a cohort study.

## MEMBER LIST OF THE JACC STUDY GROUP

The present investigators involved, with the co-authorship of this paper, in the JACC Study and their affiliations are as follows: Dr. Akiko Tamakoshi (present chairman of the study group), Nagoya University Graduate School of Medicine; Dr. Mitsuru Mori, Sapporo Medical University School of Medicine; Dr. Yutaka Motohashi, Akita University School of Medicine; Dr. Ichiro Tsuji, Tohoku University Graduate School of Medicine; Dr. Yosikazu Nakamura, Jichi Medical School; Dr. Hiroyasu Iso, Institute of Community Medicine, University of Tsukuba; Dr. Haruo Mikami, Chiba Cancer Center; Dr. Yutaka Inaba, Juntendo University School of Medicine; Dr. Yoshiharu Hoshiyama, Showa University School of Medicine; Dr. Hiroshi Suzuki, Niigata University School of Medicine; Dr. Hiroyuki Shimizu, Gifu University School of Medicine; Dr. Hideaki Toyoshima, Nagoya University Graduate School of Medicine; Dr. Shinkan Tokudome, Nagoya City University Graduate School of Medical Science; Dr. Yoshinori Ito, Fujita Health University School of Health Sciences; Dr. Shuji Hashimoto, Fujita Health University School of Medicine; Dr. Shogo Kikuchi, Aichi Medical University School of Medicine; Dr. Akio Koizumi, Graduate School of Medicine and Faculty of Medicine, Kyoto University; Dr. Takashi Kawamura, Kyoto University Center for Student Health; Dr. Yoshiyuki Watanabe, Kyoto Prefectural University of Medicine Graduate School of Medical Science; Dr. Tsuneharu Miki, Kyoto Prefectural University of Medicine Graduate School of Medical Science; Dr. Chigusa Date, Faculty of Human Environmental Sciences, Mukogawa Women’s University ; Dr. Kiyomi Sakata, Wakayama Medical University; Dr. Takayuki Nose, Tottori University Faculty of Medicine; Dr. Norihiko Hayakawa, Research Institute for Radiation Biology and Medicine, Hiroshima University; Dr. Takesumi Yoshimura, Institute of Industrial Ecological Sciences, University of Occupational and Environmental Health, Japan; Dr. Akira Shibata, Kurume University School of Medicine; Dr. Naoyuki Okamoto, Kanagawa Cancer Center; Dr. Hideo Shio, Moriyama Municipal Hospital; Dr. Yoshiyuki Ohno, Asahi Rosai Hospital; Dr. Tomoyuki Kitagawa, Cancer Institute of the Japanese Foundation for Cancer Research; Dr. Toshio Kuroki, Gifu University; and Dr. Kazuo Tajima, Aichi Cancer Center Research Institute.
